# Intestinal Multi-Target Mechanisms of Natural Active Substances in Hyperuricemia Alleviation: Recent Progress

**DOI:** 10.3390/nu18060997

**Published:** 2026-03-20

**Authors:** Ying Chen, Ziling Pan, Hongyan Li, Ke Wang, Yousheng Wang

**Affiliations:** 1 Key Laboratory of Geriatric Nutrition and Health, Beijing Technology and Business University, Beijing 100080, China; chenying@btbu.edu.cn (Y.C.); 15823865129@163.com (Z.P.); 13231056672@163.com (H.L.); wangke9509@163.com (K.W.); 2 Institute of Modern Fermentation Engineering and Future Foods, Guangxi University, Nanning 530004, China; 3 Joint Research Institute of Future Foods and Comprehensive Health Industries, Guangxi University & Guangxi Zhongbo Nuocheng Biotechnology Co., Ltd., Nanning 530004, China; 4 College of Light Industry and Food Engineering, Guangxi University, Nanning 530004, China

**Keywords:** hyperuricemia, intestinal microbiota, barrier function, transporters, natural active substances

## Abstract

Hyperuricemia, a common metabolic disorder characterized by elevated serum uric acid (UA) levels, can lead to severe complications such as gout and renal impairment. Conventional therapies, while effective, are frequently accompanied by significant adverse effects, underscoring the urgent need for safer therapeutic alternatives. Recent evidence identifies the intestine as a novel, pivotal regulator of UA homeostasis, presenting a promising therapeutic axis. This review delineates the intestinal mechanisms governing UA regulation and evaluates the therapeutic potential of natural active substances that target these pathways. We conducted a comprehensive review of recent preclinical studies focusing on intestinal mechanisms involved in UA metabolism, including the roles of gut microbiota, urate transport proteins, intestinal barrier function, and inflammation. Studies evaluating natural active substances—such as polyphenols, polysaccharides, peptides, and plant extracts—were systematically analyzed for their effects on gut-mediated UA regulation. Natural active substances have been shown to effectively alleviate hyperuricemia by modulating gut microbiota, enhancing UA intestinal excretion, reinforcing intestinal barrier function, and suppressing inflammatory pathways. Collectively, these findings demonstrate the multi-target efficacy of natural active substances within the intestines, offering a promising therapeutic strategy that warrants further investigation into nutrition-based intestinal interventions and novel pharmacological treatments for hyperuricemia.

## 1. Introduction

Uric acid (UA), the final metabolite of purine degradation, is tightly regulated by hepatic production, renal excretion, and intestinal elimination [1,2]. Hyperuricemia (HUA) arises when this balance is disrupted, leading to serum UA concentrations exceeding 7.0 mg/dL in men and 6.0 mg/dL in women, primarily due to UA overproduction and/or underexcretion [1,3]. Globally, the prevalence of HUA has increased markedly over the past two decades, making it the second most common metabolic disorder after type 2 diabetes mellitus [4,5]. This rise is largely driven by modern dietary and lifestyle habits, such as high intake of purine-rich foods (e.g., red meat, seafood, beer) and physical inactivity, both of which exacerbate purine metabolism and impair UA clearance [6,7]. In addition to these environmental factors, genetic predisposition plays a substantial role in HUA susceptibility, with heritability estimates for serum urate concentrations ranging from 40% to 70% across populations. Large-scale genome-wide association studies have identified 351 loci associated with urate levels, including urate transporter genes and metabolic regulators such as glucokinase regulator (GCKR), underscoring the polygenic architecture of urate homeostasis [8,9]. Building upon this genetic architecture, Mendelian randomization studies have further established that genetically elevated urate levels are causally linked to gout risk, independent of confounders. After accounting for pleiotropic effects using MR Egger regression, this causal relationship remained robust, whereas associations with cardiovascular and metabolic outcomes were largely attributable to pleiotropy rather than a direct causality relationship [10].

**Figure 1:**
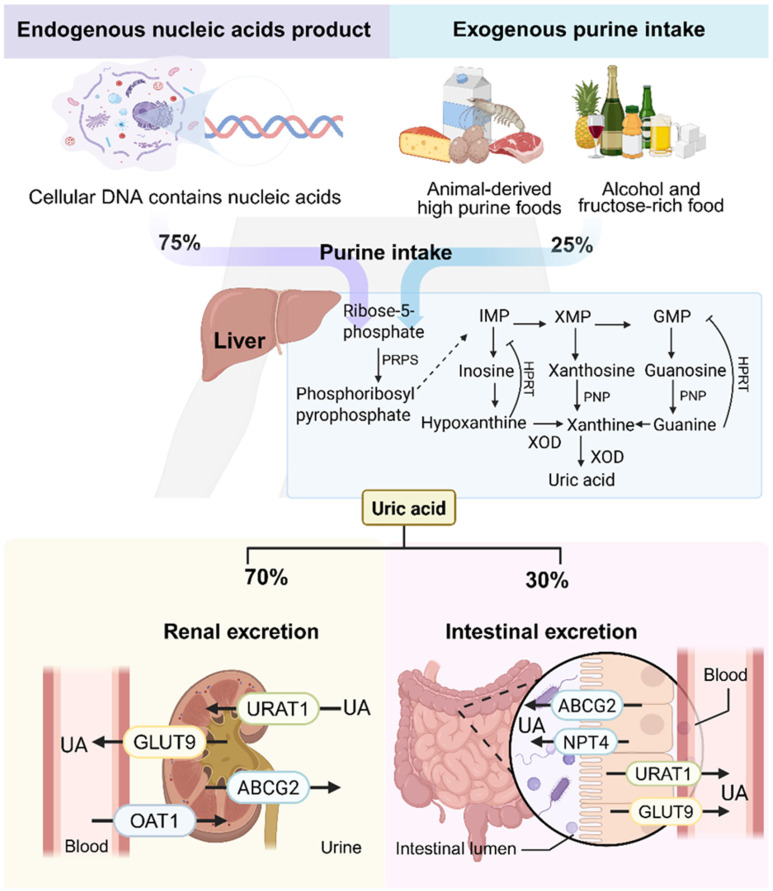
Integrated Pathways of Purine Metabolism: Endogenous Synthesis, Exogenous Intake, and Excretion. Abbreviations: IMP, inosine monophosphate; GMP, guanosine monophosphate; XMP, xanthosine monophosphate; PNP, purine nucleotide phosphorylase; PRPS, phosphoribosylpyrophosphate synthetase; XOD, xanthine oxidase; UA, uric acid; ABCG2, ATP-binding cassette subfamily G member 2; GLUT9, glucose transporter 9 (SLC2A9); URAT1, urate transporter 1 (SLC22A12); OAT, organic anion transporter; NPT4, sodium phosphate transporter 4. Solid arrows indicate single-step reactions. Dashed arrows indicate multi-step reactions.

The imbalance between UA production and elimination underpins the development of HUA. Excess UA production can result from heightened endogenous purine turnover or increased dietary purine intake [11,12]. Notably, endogenous purines, derived from nucleic acid metabolism, contribute approximately 75% of total body UA [13], while exogenous sources include purine-rich foods such as meats, shellfish, soy products, wheat, and high-fructose items [14,15]. Xanthine oxidase (XOD) and adenosine deaminase (ADA) are pivotal enzymes in UA biosynthesis. XOD, predominantly expressed in the liver and small intestine, catalyzes the oxidation of hypoxanthine and xanthine into UA. ADA facilitates the deamination of adenosine monophosphate (AMP) to inosine, which is subsequently converted to hypoxanthine and then to UA via XOD and nucleoside phosphorylase [16]. As the end product of purine metabolism, UA is primarily excreted through the kidneys (70%), with the remaining 30% eliminated through the intestinal tract (Figure 1) [17,18].

Current pharmacologic treatments for HUA fall into three major categories: inhibitors of UA synthesis (e.g., allopurinol, febuxostat); uricosuric agents that enhance renal UA excretion (e.g., probenecid, benzbromarone); and recombinant enzymes promoting UA degradation (e.g., pegloticase) [19,20]. However, these therapies are often limited by adverse effects, including severe hypersensitivity reactions, nephrotoxicity, hepatotoxicity, and cardiovascular complications [21]. Moreover, as most UA-lowering agents act primarily on the kidneys, they may impose an additional burden on patients with underlying renal dysfunction.

The intestinal tract has emerged as a central player in maintaining human uric acid homeostasis, exerting a regulatory influence that extends well beyond its classical perception as a simple conduit. It facilitates a significant extrarenal clearance route, accounting for an estimated one-third of total urate disposition. This physiological process is critically dependent on the function of specific efflux transporters, notably ABCG2, which mediates the secretion of urate into the intestinal lumen. Furthermore, the vast and complex gut microbiota is deeply integrated into urate metabolism, functioning as both a direct participant and a systemic regulator. A substantial consortium of commensal bacteria, including select species such as *Lactobacillus* spp. and *Akkermansia muciniphila*, possesses the enzymatic machinery to catabolize luminal urate into highly soluble compounds like allantoin, thereby providing a crucial mechanism for non-renal urate disposal. Conversely, a dysbiotic microbial state is strongly implicated in the etiology of hyperuricemia. The detrimental impact of dysbiosis is twofold: it involves a reduction in the collective uricolytic capacity of the microbiota and, perhaps more consequentially, a profound alteration in the production of key immunomodulatory metabolites, such as the short-chain fatty acid butyrate. Recent investigations have illuminated the multifaceted role of butyrate, revealing its capacity to both inhibit xanthine oxidase (XOD) to curtail urate synthesis, upregulate urate transporters in both enteric and renal tissues to enhance excretion, and fortify the intestinal epithelial barrier to mitigate systemic inflammation triggered by urate crystal deposition. Concurrently, compromised intestinal barrier integrity and increased permeability exacerbate systemic inflammation, creating a pathological loop that sustains the progression of HUA [22]. In conclusion, the intestinal tract, as the main pathway for extrarenal uric acid excretion, has great potential for uric acid regulation. It is expected to become a safer and more effective target for uric acid-lowering drugs and has become a new research hotspot for the treatment of hyperuricemia. This review comprehensively examines the intestinal mechanisms involved in HUA, including the roles of gut microbiota, uric acid transporter proteins, intestinal epithelial barrier function, and inflammation.

Natural active substances are compounds derived from natural sources, such as plants, animals, or microorganisms. Unlike conventional pharmacotherapies, natural bioactive compounds often exert multitargeted actions with fewer side effects, thereby constituting a crucial component currently employed in the treatment of HUA. These compounds show potential in modulating the gut–urate axis by restoring microbial balance, regulating transporter expression, enhancing barrier function, and attenuating inflammation [23,24]. Here, we summarize recent preclinical findings on natural substances that ameliorate hyperuricemia via intestinal mechanisms. This review aims to provide new insights into the intestinal pathophysiology of hyperuricemia and to guide the development of safer, intestine-targeted therapies using natural active compounds.

## 2. The Role of the Intestine in Hyperuricemia

The intestine plays a multifaceted role in UA homeostasis, functioning both as a major site of extrarenal UA excretion and as a key interface in systemic metabolic regulation. Emerging evidence highlights four interrelated mechanisms by which intestinal dysfunction contributes to hyperuricemia: (1) gut microbiota dysbiosis and altered microbial metabolites; (2) impaired UA transporter function; (3) disruption of intestinal barrier integrity; and (4) chronic intestinal inflammation. These disruptions collectively disturb UA synthesis, metabolism, and clearance, ultimately promoting pathological UA accumulation.

### 2.1. Dysbiosis of Intestinal Flora and Metabolic Disorder

Gut microbiota dysbiosis is a hallmark of HUA, marked by an increase in pathogenic taxa, a decline in beneficial commensals, and loss of microbial network stability [25]. Functional modules within gut microbes—especially those governing purine catabolism—are central to UA regulation. Metagenomic analyses demonstrate that purine metabolism is a phylogenetically conserved trait across microbial genera such as *Bacteroides*, *Clostridium*, and *Pseudomonas*, that harbor core gene clusters (e.g., *dpaL*, *hydA*, *ssnA*, *ygeY*, *xdhD*) encoding enzymes for anaerobic purine and UA degradation [26]. Notably, genera such as *Lactobacillus* and *Pseudomonas* express enzymes like uricase, allantoinase, and allantoicase that sequentially convert UA to 5-hydroxyisourate, allantoin, allantoic acid, and ultimately urea [27,28]. These enzymatic cascades facilitate: (i) catabolic breakdown of purines into non-absorbable metabolites; (ii) UA clearance via transporter modulation; and (iii) direct conversion of UA into soluble byproducts. For instance, *Lactobacillus* and *Pseudomonas* strains exhibit strong uricolytic capacity, and *Lactobacillus reuteri* TSR332 reduces serum UA (SUA) levels by 19% in HUA rats through targeted degradation of inosine and guanosine precursors [29].

Stable isotope tracing has identified 46 bacterial taxa—including *Actinobacillus*, *Clostridium*, and *Fusobacterium*—with active UA-degrading capabilities. Among Enterobacteriaceae, *Klebsiella* spp. show the highest uricase expression [30]. Conversely, HUA-associated microbiota are dominated by opportunistic pathogens such as *Escherichia-Shigella* and *Streptomyces*, which lack effective uricolytic pathways but display elevated XOD activity [31]. This bidirectional disruption of purine metabolism—loss of protective enzymes and gain of pro-uricogenic enzymes—underlies microbial contributions to hyperuricemia.

In addition to enzymatic pathways, microbial metabolites like short-chain fatty acids (SCFAs) and amino acids critically modulate host metabolism [32]. Butyrate enhances ABCG2-mediated UA excretion by activating PPARγ signaling and inhibiting histone deacetylases, thereby upregulating transporter expression in enterocytes [33]. Propionate reduces SUA by 22% in HUA mice through a dual mechanism: direct inhibition of hepatic XOD activity and reshaping of the luminal environment to favor colonization by *Lactobacillus* and *Bifidobacterium* [34]. These effects are amplified by SCFA-induced ATP generation in intestinal epithelial cells, fueling energy-dependent UA efflux [35]. Loss of SCFA-producing genera such as *Ruminococcus* and *Lactobacillus* disrupts ABCG2 function, while phylum-level dysbiosis—evidenced by Proteobacteria expansion (e.g., *Anaplasma*) and Bacteroidetes decline (e.g., *Rickettsia*)—undermines cross-feeding interactions essential for purine metabolism [36].

This dysbiotic environment perpetuates a self-reinforcing cycle: accumulating UA selectively inhibits acid-sensitive beneficial taxa (e.g., *Lactobacillus*) while promoting acid-tolerant pathogens (e.g., *Streptococcus*), which further elevate XOD activity and sustain hyperuricemia [37]. A comparative study of normouricemic individuals, asymptomatic HUA subjects, and gout patients revealed distinct gut microbial gene expression profiles, particularly in pathways related to purine, pyruvate, and amino acid metabolism—including glycine, phenylalanine, and tryptophan [38,39]. Glycine and aspartic acid, abundant in gout patients, act as key precursors in purine biosynthesis, thereby exacerbating UA overload [40]. In parallel, elevated plasma levels of cysteine, glutamine, phenylalanine, and threonine correlate strongly with HUA incidence, indicating amino acid dysregulation as a metabolic signature of hyperuricemia [41]. Collectively, gut-derived metabolites regulate epithelial energy metabolism and facilitate intestinal UA excretion, positioning the microbiota as both a target and mediator of hyperuricemia interventions.

### 2.2. Intestinal Barrier Damage and Increased Permeability

The intestinal barrier plays a critical role in maintaining metabolic homeostasis by enabling selective nutrient absorption while preventing the pathogen invasion. It consists primarily of the epithelial cell layer and the overlying mucus, both of which work in concert to regulate mucosal permeability and isolate the intestinal lumen. Tight junction (TJ) proteins within the epithelium form a semi-permeable seal, while the mucus layer shields epithelial cells and supports the growth of commensal microbiota. In hyperuricemia (HUA), this barrier becomes compromised due to inflammation and microbial dysbiosis, resulting in increased permeability and impaired uric acid regulation [42,43].

Experimental models support this mechanism. In *Uox*-knockout mice—a well-established model of hyperuricemia—significant intestinal abnormalities were observed, including shortened villi, mucosal edema, and mucus layer thinning. These changes are accompanied by downregulation of key TJ proteins such as ZO-1 and occludin, alongside elevated serum levels of permeability markers including diamine oxidase (DAO), D-lactic acid (D-LAC), and endotoxin [44]. Additional studies confirm that Uox(−/−) mice exhibit progressive increases in intestinal permeability, marked by reduced expression of occludin and claudin-1 and upregulation of the pro-apoptotic protein Bax, indicating both structural damage and apoptotic signaling in epithelial cells [22].

Recent evidence underscores a bidirectional relationship between microbiota dysbiosis and intestinal barrier dysfunction during HUA progression. Damage to the mucosa results in TJ contraction and internalization, widening intercellular gaps and allowing luminal contents, including bacteria and their products, to breach the barrier [45]. Some bacterial strains transiently induce TJ expression by suppressing the TLR4/NF-κB signaling pathway; however, these compensatory mechanisms are insufficient to restore long-term barrier integrity. Germ-free and antibiotic-treated animal models further demonstrate that the presence of a dysbiotic microbiota is essential for the development of epithelial damage under hyperuricemic conditions. Removal of microbial stimuli restores barrier integrity despite elevated UA levels [46]. Together, these findings suggest that intestinal barrier breakdown in HUA is primarily mediated by microbiota-induced disruption of TJs and mucus degradation, thereby perpetuating a cycle of increased permeability, microbial translocation, and systemic UA dysregulation.

### 2.3. Intestinal Inflammation and Systemic Spread

Chronic low-grade inflammation is a central pathological feature of hyperuricemia. It is driven by synergistic interactions between compromised intestinal barrier integrity and microbial dysbiosis, which facilitate the translocation of bacteria, lipopolysaccharide (LPS), and pro-inflammatory cytokines into systemic circulation, thereby amplifying inflammatory responses [36]. LPS, a potent endotoxin derived from Gram-negative bacteria, not only increases intestinal wall permeability but also stimulates massive cytokine release, fostering sustained inflammation—a defining characteristic of HUA [47]. It is crucial to note that lipopolysaccharides have been demonstrated to trigger systemic inflammatory responses by activating the Toll-like receptor 4 (TLR4) signaling pathway in proximal tubule cells. These agents directly impact renal uric acid metabolism by regulating the expression of key uric acid transporters (such as URAT1 and GLUT9) in proximal tubule cells, thereby exacerbating hyperuricemia [48]. This inflammatory cascade further disrupts epithelial barrier integrity and microbial homeostasis, forming a self-reinforcing loop. The synergistic effect of translocated bacterial components (e.g., LPS) and the depletion of microbial metabolites (e.g., SCFAs) worsens epithelial damage and stimulate systemic immune activation, linking local barrier failure to systemic inflammatory responses [49]. Multi-omics analyses reveal that HUA-associated dysbiosis leads to the depletion of anti-inflammatory taxa, such as *Faecalibacterium*, and the loss of their immunoregulatory metabolites, including butyrate, thereby impairing endogenous mucosal repair and immune tolerance [30]. Specifically, in gut-associated lymphoid tissue (GALT), dysbiosis-induced barrier dysfunction allows LPS to activate TLR4 signaling, priming NLRP3 inflammasome expression. Concurrently, MSU crystals or metabolites such as ATP provide another signal that triggers NLRP3 assembly and caspase-1 activation, leading to IL-1β and IL-18 maturation and release. Thus, intercepting NLRP3 activation in GALT represents a critical checkpoint for suppressing the inflammatory cascade of the gut–joint axis [50,51]. These observations indicate that intestinal inflammation in HUA is both a consequence of UA overload and a key driver of disease progression.

### 2.4. Imbalance of Intestinal Uric Acid Transport System

Multiple transporters in the intestinal epithelium mediate UA absorption and secretion, with ATP-binding cassette subfamily G2 (ABCG2) and glucose transporter 9 (GLUT9) being the most critical [52]. GLUT9, also known as SLC2A9, facilitates UA reabsorption into enterocytes and is highly expressed in the jejunum and ileum, predominantly localized to apical and basolateral membranes of intestinal epithelial cells [53]. It is considered a high-capacity UA transporter, and its expression levels are strongly correlated with systemic UA concentrations. Mice lacking intestinal GLUT9 exhibit elevated SUA and early-onset HUA with associated features of metabolic syndrome [54]. Additionally, single nucleotide polymorphisms (SNPs) in SLC2A9 have been linked to impaired intestinal UA excretion and elevated SUA [55].

ABCG2, a major UA efflux transporter distributed throughout the small and large intestine, mediates UA excretion into the intestinal lumen. In *Abcg2* knockout mice, intestinal UA elimination is reduced by more than 50%, with a compensatory increase in renal excretion. Conversely, nephrectomized rats with impaired renal function show upregulated ABCG2 expression in the intestine, highlighting its compensatory role in extrarenal UA clearance [56].

Genetic variants in *ABCG2* profoundly influence SUA levels by affecting transporter expression and functionality. The Q141K and Q126K variants are particularly pathogenic, causing protein misfolding, retention in the endoplasmic reticulum, and increased degradation, leading to reduced plasma membrane expression and impaired UA transport [57]. Although ABCG2 is expressed in both the kidney and intestine, defective intestinal excretion due to the Q141K mutation is believed to be a primary driver of HUA and gout. Notably, the histone deacetylase inhibitor 4-phenylbutyric acid has been shown to correct misfolded 141K ABCG2 protein and restore its function, offering a potential therapeutic strategy for hyperuricemia [58,59].

### 2.5. Inter-Mechanism Interaction Network

Experimental evidence shows that exogenous UA promotes intestinal inflammation by elevating pro-inflammatory cytokine levels, which in turn damage the epithelial barrier and exacerbate microbial imbalance. This dysbiosis increases mucosal permeability and facilitates bacterial translocation, perpetuating a chronic inflammatory state. In Uox-deficient mice, enrichment of pro-inflammatory bacteria such as *Escherichia-Shigella* activates TLR2/4/5 signaling pathways, triggering NF-κB–mediated overproduction of IL-1β and TNF-α. These cytokines not only suppress TJ proteins such as occludin and claudin-1 but also recruit immune cells, further damaging the intestinal mucosa [22].

Ultimately, gut dysbiosis and epithelial disruption initiate and sustain an inflammatory loop mediated by TLR/NF-κB activation in response to translocated LPS. This loop accelerates TJ degradation, promotes systemic inflammation, and exacerbates hyperuricemia. Understanding this gut–inflammation axis is essential for the development of targeted interventions that restore barrier function, rebalance the microbiota, and mitigate chronic inflammation in hyperuricemia.

## 3. Natural Products Targeting the Gut in HUA

**Figure 2:**
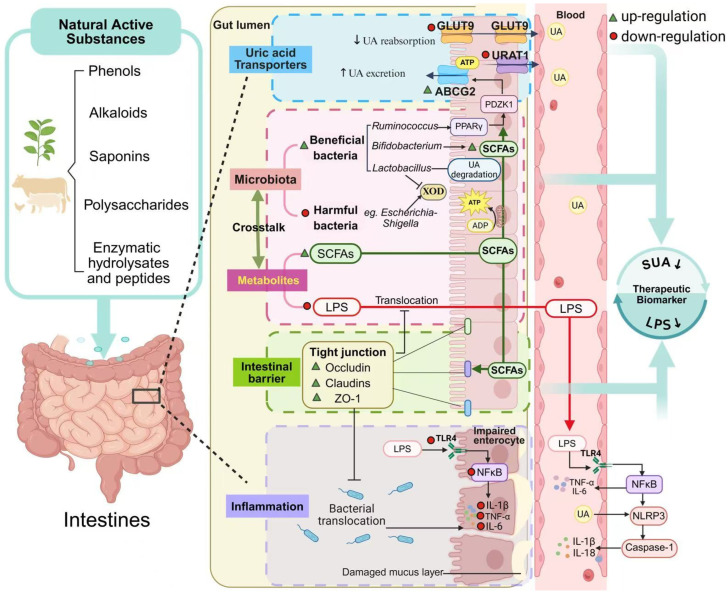
Mechanisms of natural active substances in alleviating hyperuricemia via intestinal regulation. Abbreviations: UA, uric acid; XOD, xanthine oxidase; SCFAs, short-chain fatty acids; ABCG2, ATP-binding cassette subfamily G member 2; GLUT9, glucose transporter 9; ZO-1, zonula occludens-1; LPS, lipopolysaccharide; PPAR, peroxisome proliferator-activated receptor; TLR4, Toll-like receptor 4; NF-κB, nuclear factor kappa-light-chain-enhancer of activated B cells; NLRP3, NOD-like receptor protein 3; IL-1β, interleukin-1 beta; IL-6, interleukin-6; TNF-α, tumor necrosis factor-alpha.

In recent years, increasing attention has been directed toward the therapeutic potential of natural products in regulating UA metabolism. Due to their multi-component and multi-target characteristics, natural bioactives are ideally suited for intervening in the aforementioned complex intestinal mechanistic network. Emerging mechanistic studies reveal that natural compounds can modulate gut microbiota, triggering a networked regulatory mechanism involving host metabolism, intestinal barrier function, inflammation, and transporter activity [60]. To provide a comprehensive and systematic overview, we conducted a literature search in major scientific databases (e.g., PubMed, Web of Science, Scopus) using a combination of keywords including “hyperuricemia,” “gut microbiota,” “intestinal barrier,” “urate transporters,” and “natural products”. Studies published within approximately the last five years (2020–2025) in peer-reviewed journals were considered for inclusion. The inclusion criteria were as follows: (1) original research articles; (2) focusing on the effects of natural active substances on HUA; (3) studies focusing on intestinal mechanisms, reporting at least one of the following: gut microbiota, barrier function, inflammation, or urate transporters; and (4) studies employing in vivo animal models of hyperuricemia. We focus particularly on preclinical studies, as they provide critical mechanistic insights into the multi-target intestinal pathways involved in uric acid regulation, such as microbiota modulation, barrier function, UA transporters expression, and inflammatory signaling—mechanisms that are difficult to elucidate in human trials. Furthermore, animal models allow for controlled interventions and tissue-level analyses, providing an important foundation for understanding the pharmacological effects of natural compounds prior to clinical translation. We anticipate that this review will aid in understanding the role of natural products in the treatment of HUA, providing a theoretical basis for the future development of urate-reducing drugs targeting the intestine (Figure 2).

### 3.1. Regulation of Intestinal Flora and Metabolism

Recently, a series of studies have explored the potential therapeutic effects of natural products as treatments for hyperuricemia, which are exerted by influencing the gut microbiota (Table 1) [61]. Some flavonoids or polyphenols in plant-derived natural products can function as antibiotics to suppress the proliferation of detrimental microorganisms [62]. Resveratrol (RES) exhibits notable anti-hyperuricemic effects by boosting intestinal UA catabolism and reshaping the gut microbiome. RES stimulates the proliferation of uricase-producing *Lactobacillus* species, reduces harmful bacteria that promote inflammation, and improves microbial function via the upregulation of purine metabolism. It concurrently suppresses renal inflammation (IL-6, IL-1β, TNF-α), partly via enrichment of *Bifidobacterium* and SCFA-producing genera. The FMT experiment revealed that fecal bacteria from the RES treatment significantly enhanced the abundance of *Ligilactobacillus* and decreased the abundance of *Eisenbergiella*, which is reportedly associated with inflammatory disorders. The results suggest that RES may improve intestinal UA metabolism by reversing intestinal microbiota disturbances [63]. A hybrid flavonoid compound, myricetin-nobiletin (MNH), improves HUA through dual targeting of gut dysbiosis and glycerophospholipid metabolism. MNH promotes the enrichment of *norank_f_Muribaculaceae* and *Bacteroides*, while suppressing *Lactobacillus* and *Limosilactobacillus*. It also restores arachidonic acid metabolism by increasing 20-hydroxy-leukotriene B4 and supports neuronal health via N-acetylaspartate upregulation—together resolving HUA-induced metabolic-inflammatory cascades [64]. Quercetin has been demonstrated to mitigate HUA by remodeling the intestinal microbiota of chickens. It showed that quercetin promotes the growth of *Lactobacillus avium* CML180, which expresses nucleoside hydrolase Nhy69—a key enzyme in purine degradation—thereby suppressing UA synthesis. The targeted regulation of gut microbiota contributes to the effect of quercetin in alleviating hyperuricemia by mediating the catabolism of nucleosides in the gastrointestinal tract [65]. Fisetin, another flavonol, alleviates HUA-related renal injury by enhancing microbiota-host metabolic crosstalk. Fisetin treatment leads to significant modification of the bacterial structure in the mouse intestine both at the genus and phylum levels (*p* < 0.05). Specifically, it restores the gut microbiota-derived tryptophan metabolite, L-kynurenine in hyperuricemic mice, and inhibits the aryl hydrocarbon receptor (AHR), thereby attenuating renal inflammation and chronic kidney disease (CKD) [66].

*Camellia japonica* Bee Pollen and Extract (CPE-E), rich in quercetin, kaempferol, and gallic acid, enhances the abundance of *Lactobacillus* and Clostridiaceae, boosting SCFA production—especially of propionic and butyric acids—which provide energy to epithelial cells and promote UA excretion [67]. Kidney tea (KT), rich in rosmarinic and ursolic acids, significantly reduces serum UA levels and ameliorates kidney injury in HUA mice (*p* < 0.05). KT modifies the gut microbial community by enriching beneficial genera (e.g., *Roseburia*, *Enterorhabdus*) and suppressing harmful ones (e.g., *Ileibacterium*, UBA1819). This remodeling is associated with normalized levels of 33 fecal and serum metabolites, including derivatives of phenylalanine, tyrosine, and tryptophan, resulting in suppressed kynurenine-driven inflammation and restoration of renal UA transporter expression [68].

Saponins, amphiphilic glycosides with both hydrophilic and lipophilic moieties, exert multifactorial anti-HUA effects. Astragaloside IV (AST), a triterpenoid saponin from *Astragalus membranaceus*, mitigates the accumulation of febuxostat and corrects urea metabolism imbalances in hyperuricemic nephropathy rats. It decreases harmful genera such as *Eubacterium*, *Parabacteroides*, *Ruminococcus*, and *Clostridium*, while reprogramming the hepatic-intestinal ammonia cycle. Notably, AST has very low oral bioavailability (<2.5%), which would typically limit the systemic efficacy. However, oral (but not intraperitoneal) AST administration significantly improved the pharmacokinetics of febuxostat in HN rats, suggesting that the unabsorbed fraction of AST interacts directly with the gut microbiota in the intestine, inducing changes in the intestinal environment that mediate its therapeutic effects. This finding exemplifies how low bioavailability can be advantageous for gut-targeted interventions [69]. Ultrafine powder of *A. membranaceus* (AMUP), enriched in saponins, reconstitutes gut microbial composition, stabilizes purine metabolism, and enriches beneficial microbes like *Clostridium* and Trichosporonaceae (*p* < 0.05). Drug metabolism analysis demonstrated that flavonoid constituents are distributed in the blood, whereas saponins remain in the intestine. This differential distribution suggests that flavonoids exert systemic therapeutic effects after absorption, while saponins—being poorly absorbed—play a specific role in regulating the community structure of the intestinal flora. Notably, high-dose AMUP may exhibit certain toxic side effects, as its microbiome profile differed significantly from that of healthy rats, indicating that dose optimization is critical for therapeutic application [70]. Rare ginsenosides (RGS) also demonstrate preventive and therapeutic potential in HUA by enhancing sphingolipid and pyrimidine metabolic pathways. RGS restores the Firmicutes/Bacteroidetes (F/B) ratio and boosts microbial diversity (*p* < 0.05), contributing to improved intestinal and systemic metabolic profiles [71].

Related research also showed that some polysaccharides in natural products can serve as prebiotics, improving the structure of intestinal microbial communities or promoting the growth of beneficial bacteria, thereby regulating the host’s uric acid metabolism. *Alpinia oxyphylla* fruit polysaccharides (AFP) is a heteropolysaccharide extracted from the fruit of *Alpinia oxyphylla*. AFP restored the gut microbiota in hyperuricemic mice, increasing the abundance of *Prevotella* and *Ruminococcus* while suppressing the abundance of *Bacteroides*, *Parabacteroides*, *Helicobacter*, and *Flexispira*. Notably, the abundance of *Ruminococcus* is inversely related to the levels of IL-6 and IL-1β, while the presence of *Bacteroides* and *Helicobacter* correlates positively with renal LPS concentrations. AFP also reduced serum endotoxin levels and showed a trend towards upregulating intestinal ABCG2 mRNA expression, though this did not reach statistical significance (*p* > 0.05). These findings suggest that AFP contributes to the restoration of a balanced intestinal environment and the suppression of inflammatory responses [72]. Levan, a microbial exopolysaccharide fructan, improves lipid and glycerophospholipid metabolism and boosts immunity in hyperuricemic rats by restoring the composition of intestinal microorganisms and metabolites in HUA rats. It increases beneficial taxa such as Muribaculaceae, *Faecalibaculum*, *Bifidobacterium*, and *Lactobacillus*, while reducing pathogenic *Escherichia_Shigella* and *Proteus* [73]. Insoluble fiber from barley leaves (BL) enhances SCFA-producing *Bacteroides* species (e.g., *B. mimicus*, *B. isotopicus*, *B. eisenbergius*) and increases SCFAs levels. Of interest, supplementation with sodium acetate, propionate, and butyrate in hyperuricemic mice can effectively reduce their serum UA levels. Furthermore, SCFAs dose-dependently inhibit URAT1 and GLUT9 in vitro (*p* < 0.05). The results indicate that BL and its metabolite SCFAs may be potential candidates for relieving HUA [74]. Polysaccharides derived from brown algae, such as Guluronate oligosaccharides (GOS), reshape the gut microbial landscape by enriching beneficial taxa (e.g., Lachnospiraceae_UCG_006, *Ruminococcus*) and reducing harmful bacteria (e.g., Bilophila, Tuzzerella) and producing short-chain fatty acids. GOS also downregulates intestinal UA reabsorption transporters GLUT9 and SMCT (*p* < 0.05), facilitating UA excretion. Study limitations include the absence of mechanistic validation using antibiotics or germ-free models to confirm gut microbiota dependency, and the lack of identification of specific SCFA transporters or receptors involved [75]. *Sporisorium reiliana* polysaccharides alleviate gut dysbiosis by reducing *Mycobacterium anisopliae* abundance and downregulating genes associated with glycolysis and purine metabolism, thus limiting UA synthesis [76].

Alkaloids are nitrogen-containing secondary metabolites found abundantly in plants; nuciferine, for example, significantly alleviates HUA. Integrated ^1^H NMR/LC-MS metabolomics and microbiota profiling reveal that nuciferine reshapes gut microbiota and rebalances host-microbe co-metabolism, ameliorating systemic metabolic dysfunction [77]. Sulforaphane (SFN), an isothiocyanate from cruciferous vegetables, reverses HUA-associated metabolic disturbances by enriching *Clostridium* spp. (e.g., *C. perfringens*, *C. azureum*, and *C. lactis*) and enhancing amino acid methylation. SFN also activates nuclear factor erythroid 2-related factor 2 (Nrf2) via epigenetic pathways, reducing renal oxidative stress through gut microbiota–metabolite association. Unlike many phytochemicals with limited bioavailability (typically 1–8%), SFN exhibits approximately 80% bioavailability due to its small size and lipophilic nature. This high bioavailability, combined with its isothiocyanate structure, enables SFN to engage in multifaceted interactions with both the gut microbiota and host tissues. Study limitations include the use of bioactivated glucoraphanin rather than pure SFN, and the need for experimental validation of bioinformatics-based speculations regarding microbiota-epigenome interactions [78]. Two novel hexapeptides (GPAGPR and GPSGRP), derived from *Astragalus japonicus* hydrolysate, reduced serum UA in mice. These peptides enhanced gut microbial diversity and enriched taxa associated with SCFA production, bile acid metabolism, and tryptophan catabolism. The modified microbiota and its metabolites are proposed to mediate systemic anti-inflammatory and metabolic effects, contributing to uric acid reduction. The peptides thus function as effective microbiota-modulating agents with potential for managing hyperuricemia [79]. Tuna meat oligopeptides (TMOP) alleviated hyperuricemia by correcting gut microbiota imbalances and increasing SCFA production. Fecal microbiota transplantation from TMOP-treated mice successfully replicated these benefits, confirming the microbiota-mediated mechanism. However, FMT failed to significantly improve serum creatinine and urea nitrogen levels, indicating that TMOP’s renoprotective effects may involve additional mechanisms beyond microbiota modulation [80].

### 3.2. Regulation of Intestinal Barrier Function and Inflammation

The intestinal barrier is essential for human health, and it constitutes the interface between the external and internal environments of the body. When intestinal mucosa is damaged, UA can readily traverse from the intestinal lumen into the systemic circulation, leading to hyperuricemia. Concurrently, the compromised barrier increases translocation of pathogen-associated molecular patterns and bacterial LPS, which could further cause gut dysbiosis and extensive intestinal inflammation, aggravating the development of hyperuricemia and gout. Recent studies have shown that some natural products ameliorate hyperuricemia by targeting the intestinal barrier function and inflammation.

Curcumin, the primary polyphenol in turmeric, maintains intestinal barrier integrity and lowers the circulating levels of LPS in hyperuricemic rats. The expression of tight junction proteins (ZO-1, occludin, and claudin-1) was significantly decreased in the model group (*p* < 0.001), indicating that the intestinal barrier had been damaged. The intestinal barrier dysfunction increases intestinal permeability and results in the translocation of conditionally pathogenic bacteria and LPS into the blood circulation, which causes severe metabolic endotoxemia and systemic inflammation, and in turn exacerbates renal damage. Curcumin treatment strengthens the intestinal barrier via upregulation of ZO-1 and occludin (*p* < 0.001) and alleviates metabolic endotoxemia, which prevent intestinal bacterial translocation and reduce the inflammatory response, thus indirectly protecting the renal function [81]. *Torreya grandis* seed extract induces beneficial microbial shifts—enhancing *Enterorhabdus*, Muribaculaceae, *Marvinbryantia*, and *Blautia*—which upregulate TJ proteins (e.g., ZO-1, occludin) and reduce inflammatory cytokine levels. Limitations of this study include the testing of only a single concentration and the need for further characterization of bioactive components beyond flavonoids and polyphenols [82].

Fucoidan, a marine polysaccharide, attenuates HUA-induced renal injury by enhancing intestinal barrier integrity, modulating gut microbiota, increasing butyric acid production, and activating the AMPK/AKT/CREB pathway to upregulate ileum ABCG2, thus promoting UA excretion [83]. Chicory also upregulates the tight junction proteins such as ZO-1 and occludin (*p* < 0.05), repairs mucosal damage, inhibits intestinal permeability, and suppresses inflammation in animal models, markedly reducing hyperuricemia-induced cytokine and LPS levels (*p* < 0.05) [84]. Similarly, extracts of *Dendrobium candidum* leaves inhibited hepatic XOD and ADA activities and suppressed the TLR4/NF-κB signaling pathway in both kidney and liver. Additionally, DLE improved intestinal morphology by increasing villus height and the villus height/crypt depth ratio, suggesting enhanced intestinal barrier function. However, the study did not include gut microbiota analysis, and whether the intestinal protective effects involve modulation of the gut microbiota-LPS-TLRs/NF-κB axis remains to be confirmed [85]. Plantaginis Semen polysaccharides (PSP) mitigate HUA by repairing intestinal and renal damage, restoring mucosal barrier function, inhibiting LPS translocation, and rebalancing microbial and metabolic profiles in HUA rats [86].

Chlorogenic acid (CGA), a widely distributed phenylpropanoid, improves hyperuricemic profiles by enriching SCFA-producing bacteria such as *Bifidobacterium* and *Lactobacillus* and correcting purine and glutamate metabolism dysregulation. CGA also reduces serum trimethylamine N-Oxide (TMAO) by inhibiting gut microbial choline metabolism, lowers intestinal pro-inflammatory cytokines (IL-1β, IL-6), and reinforces barrier integrity by upregulating ZO-1 and occludin [87].

Leech Poecilobdella manillensis total protein (LTP) increased beneficial genera (e.g., *Prevotella*, *Akkermansia*) and suppressed harmful bacteria, thereby regulating serum metabolites in sphingolipid and galactose pathways. These metabolic shifts correlated with key microbiota (e.g., *Bacteroides*, *Prevotella*) and facilitated uric acid reduction. Additionally, LTP enhanced the expression of intestinal tight junction proteins (*p* < 0.05), indicating improved barrier function. Overall, LTP exerts its anti-hyperuricemic effects primarily through gut microbiota remodeling and host metabolic reprogramming [88].

Allicin, the primary bioactive compound in garlic, alleviates gouty arthritis in rats by regulating the gut–joint axis. Treatment with allicin significantly reduced joint swelling, serum uric acid, and inflammatory cytokines (IL-1β, TNF-α), and restored gut microbiota balance while increasing butyric acid production. The therapeutic effect was confirmed by fecal microbiota transplantation, and in vitro studies demonstrated that butyric acid directly inhibited MSU-induced ROS production and NLRP3 inflammasome activation in fibroblast-like synoviocytes [89] (Table 2).

### 3.3. Regulation of Intestinal Uric Acid Transporters

The urate transporter proteins GLUT9, URAT1, and ABCG2 also play important roles in UA excretion in the intestine. Eupatilin, a flavonoid from *Artemisia* species, enhances ABCG2-mediated intestinal UA excretion and inhibits GLUT9 and URAT1-mediated UA reabsorption in the ileum [31]. Mangiferin promotes the intestinal secretion of endogenous urate in in situ intestinal closed loops in mice, and inhibits the absorption of exogenous uric acid perfused into the intestinal loops in rats. It facilitates endogenous UA elimination by upregulating ABCG2 and suppressing GLUT9 in the intestine in a dose-dependent manner, which might be an important mechanism underlying mangiferin-induced reductions in serum uric acid levels (*p* < 0.05) [90]. Berberine, a botanical alkaloid originally isolated from the Chinese herb Coptis chinensis, is known to possess antimicrobial activity, which is commonly used for treating gastrointestinal inflammatory diseases. One study found that berberine could decrease the levels of serum and fecal UA. A mechanism study showed that berberine can activate and improve the activity of the intestinal transporter ABCG2, and increase its expression in the intestinal tract, meaning that more UA in the blood could be transported to the intestinal tract. However, berberine’s minimal effect on urinary uric acid excretion is consistent with its primary intestinal retention and limited systemic absorption [91]. Dioscin, a steroidal saponin, indirectly lowers UA levels via its active metabolites—diosgenin and tiogenin—which enhance ABCG2-mediated UA excretion. Notably, dioscin itself is inactive in HCT116 cells, whereas its metabolites significantly enhance UA efflux—a response abolished upon ABCG2 inhibition [92]. Ferulic acid (FA) ameliorates HUA through dual mechanisms: it enhances ABCG2 expression (*p* < 0.05) and reduces *Slc2a9* (GLUT9) (*p* < 0.05) and *Slc22a13* (OAT10) (*p* < 0.05) expression in the small intestine, thereby promoting UA excretion. Simultaneously, FA inhibits the TLR4/NF-κB inflammatory axis, mitigates oxidative stress in renal tissues, and rebalances gut microbiota by increasing *Lactobacillus* and *Ruminococcus* while reducing *Bacteroides*. However, the specific anti-hyperuricemic mechanisms of FA remain to be fully clarified. Future studies employing fecal microbiota transplantation are needed to verify whether FA’s beneficial effects are directly mediated by the intestinal flora. Further investigation is required to determine whether FA directly modulates urate transporters through molecular interactions [93] (Table 3).

### 3.4. Synergistic Action of Multiple Targets

The human intestine harbors a dense and diverse community of microorganisms that, together with the intestinal mucosal barrier, metabolites, and immune system, constitutes a complex and dynamic ecosystem. This ecosystem is now recognized as a critical metabolic organ due to its pivotal role in regulating host metabolism. Recent studies have demonstrated a strong association between gut microecology and the pathogenesis of gout and hyperuricemia. Mechanistic studies suggest this connection is multifactorial, involving altered microbial degradation of purines, compromised intestinal barrier integrity leading to systemic inflammation, and the modulation of host inflammatory responses by specific microbial metabolites like short-chain fatty acids. Natural bioactives always exhibit multi-target synergy in hyperuricemia treatment, which may effectively act on multiple mechanisms within the gut microecology, resulting in a more effective reduction of uric acid.

For example, inulin, a type of fermentable dietary fiber, can effectively alleviate hyperuricemia in Uox-knockout (KO) mice. In KO mice, the expressions of tight junction proteins (occludin and ZO-1) decreases, and intestinal permeability increases. Guo et al. reported that 9.5 g/kg inulin treatment prevented hyperuricemia-induced barrier damage by enhancing the expression levels of occludin and ZO-1 in the intestinal epithelium. The intestinal mucosa intact intercellular junctions form the basis for segregation of microbes and endotoxin, thereby preventing inflammation-related conditions in the intestine. It was revealed that the levels of inflammatory cytokines and the LPS were remarkably higher in the KO group than those in the WT group, indicating systemic inflammation of hyperuricemic mice, but inulin treatment ameliorated inflammation in KO mice. It has been reported that the expression level of ABCG2 is significantly decreased during inflammation and negatively correlated with impaired intestinal barrier. Additionally, inulin supplementation led to a significant increase in intestinal expression of ABCG2 in KO mice. The results support an effective role for inulin in the maintenance of intestinal epithelial barrier integrity and intestinal UA excretion in hyperuricemic mice. In addition, inulin supplementation enhances microbial diversity and remarkably increases the relative abundance of beneficial bacteria, involving *Bifidobacterium* and *Parasutterella*, as well as the production of SCFAs [94]. Therefore, inulin might reduce host UA levels through multi-target synergistic effects in the intestine.

Oleanolic acid (OA), a pentacyclic triterpenoid, alleviates HUA by restoring gut microbial balance, increasing SCFA production, and reinforcing intestinal barrier integrity through ZO-1, claudin-1, and occludin upregulation (*p* < 0.05). OA also increases the expression of intestinal ABCG2 protein (*p* < 0.05) and decreases the expression of intestinal GLUT9 protein (*p* < 0.05). Fecal microbiota transplantation (FMT) from OA-treated donors confirmed the causal role of microbiota in mediating OA’s effects, as recipients exhibited reduced serum and urinary UA levels, attenuated renal inflammation, normalized transporter expression, and improved gut permeability. Notably, OA exhibits very low oral bioavailability (approximately 0.7% in rats) due to its poor water solubility. This characteristic, which would typically be considered a limitation, may actually represent a therapeutic advantage for gut-targeted interventions, as it allows OA to remain in the digestive tract and interact directly with the gut microbiota to exert its beneficial effects. Future studies using germ-free mice could further establish the crucial role of microbiota in OA’s anti-hyperuricemic activity [95].

Protease hydrolysates and bioactive peptides have emerged as promising anti-hyperuricemic agents owing to their multi-target activity, high bioavailability, and low toxicity. The tripeptide Pro-Glu-Trp (PEW), derived from whey protein, exhibits XOD inhibitory activity and a UA-lowering effect. Qi et al. further investigate the impact of PEW on alleviating HUA in rats from the perspective of intestine. They found that PEW inhibits the XOD activity in the serum, jejunum, and ileum, ameliorates intestinal morphology changes and oxidative stress, and upregulates the expression of ABCG2 and GLUT9 in the small intestine (*p* < 0.001). PEW reverses gut microbiota dysbiosis by decreasing the abundance of harmful bacteria (e.g., *Bacteroides*, *Alloprevotella*, and *Desulfovibrio*) and increasing the abundance of beneficial microbes (e.g., Muribaculaceae, *Lactobacillus*, and *Ruminococcus*), and elevates the concentration of SCFAs (*p* < 0.005). PEW upregulated the expression of occludin and ZO-1 (*p* < 0.01), and decreased serum IL-1β, IL-6, and TNF-α levels (*p* < 0.001) [96]. In conclusion, PEW is believed to alleviate hyperuricemia through multiple mechanisms, including intestinal UA transport, gut microbiota, and intestinal barrier (Table 4).

## 4. Conclusions and Perspective

Hyperuricemia is an escalating global health concern, driven largely by modern dietary patterns and sedentary lifestyles. While xanthine oxidase inhibitors remain standard therapy, their long-term application is limited by the toxicity and their narrow focus on renal UA clearance. Recent insights highlight the gut as a pivotal regulatory hub for UA metabolism. Natural bioactives—including polyphenols, alkaloids, polysaccharides, and enzymatic hydrolysates—demonstrate multi-target efficacy by remodeling gut microbiota, enhancing ABCG2-mediated intestinal UA efflux, and mitigating local inflammation.

Despite these promising preclinical findings, the translation of natural active substances into clinical practice faces substantial hurdles. Currently, large-scale, definitive human clinical trials specifically designed to evaluate these compounds for hyperuricemia management remain remarkably limited. The existing evidence base is predominantly derived from animal models and in vitro studies. While some small-scale clinical observations exist, these are typically constrained by small sample sizes, short follow-up durations, and inadequate control of confounding variables, rendering the evidence level insufficient to support widespread clinical adoption [97].

Several fundamental obstacles underlie this translational gap. First, a significant proportion of naturally occurring bioactive compounds exhibit inadequate solubility, constrained intestinal absorption, and substantial first-pass metabolism, consequently engendering diminished bioavailability within the body and potentially impeding their capacity to attain therapeutic concentrations in humans. Paradoxically, it is important to note that limited intestinal absorption may also confer certain advantages. Secondly, substantial variations exist in preclinical studies regarding dosing regimens, animal models, and endpoint measurements. This has resulted in limited available data that are insufficient to support the establishment of standardized administration protocols. Moreover, hyperuricemia is a chronic metabolic disorder necessitating long-term management, whereas extant research predominantly focuses on short-term interventions. Consequently, the safety of prolonged administration remains uncertain at present.

Future research must prioritize elucidating the structural and functional basis of these bioactives through integrative approaches combining computational modeling and high-throughput experimental platforms. Advanced multi-omics tools—such as metagenomics, transcriptomics, and metabolomics—will be instrumental in unraveling the molecular pathways involved and guiding the rational design of precision dietary interventions [98]. Moreover, rigorous validation in preclinical models and well-controlled human trials is essential to assess safety, efficacy, and translational potential. The development of colon-targeted delivery systems and bioavailability-enhancing formulations may further optimize therapeutic outcomes [99,100]. Recently, a chylomicron-mimicking supramolecular nanoemulsion was developed to enhance the oral bioavailability and targeted distribution of luteolin, demonstrating superior urate-lowering and nephroprotective effects in hyperuricemic animal models, highlighting the potential of advanced delivery systems for improving the efficacy of natural flavonoids against HUA [101]. Despite the advent of certain functional foods containing bioactive ingredients—including various teas and plant-based beverages—within the market, numerous products designated as containing specific active components are beset by unresolved issues pertaining to their concentration, stability, and bioavailability. This phenomenon is characterized by negligible practical efficacy. In the event of future endeavors achieving success in the transformation of active ingredients into functional foods, for instance, fermented beverages reducing urea levels or nutritionally fortified snacks, this would provide a practical strategy that would serve to bridge the gap between laboratory research and clinical application, aligning with public health nutrition initiatives [102]. By addressing these challenges and leveraging the synergistic potential of food science and biomedicine, gut-targeted strategies can be refined into effective, sustainable dietary solutions for hyperuricemia management.

## Figures and Tables

**Table 1:** Proposed mechanisms by which natural bioactives affect the gut microbiota in prevention of hyperuricemia.

Bioactive Compound	Model	Dose	Microbiota Findings	Mode of Mechanism	Reference
Resveratrol (RES)	Male C57BL/6J mice (*n* = 12/group) were fed with high-fat diet (HFD) for 8 weeks.	0.1% in diet, ≈ 100 mg/kg BW/day, p.o.	↑ *Lactobacillus* sp. ESL0791, *Lacticaseibacillus rhamnosus*; ↓ *Eisenbergiella tayi*	Decrease the expression of genes related to purine metabolism.	[63]
Myricetin and nobiletin (MNH)	Male SPF KM mice (*n* = 10/group) were fed with 15% (*w*/*w*) yeast paste diet for 9 weeks.	30 mg/kg BW/day, i.g.	↑ *norank_f_Muribaculaceae*, *Bacteroide*; ↓ *Lactobacillus*, *Limosilactobacillus*	Regulate the metabolic pathways involved in glycerophospholipid metabolism, arachidonic acid metabolism, and alanine/aspartate/glutamate metabolism.	[64]
Quercetin	Arbor Acres broilers (*n* = 10–14/group) were gavaged with yeast powder (10 g/kg) and AD (100 mg/kg) for 7 weeks; Male KM mice (*n* = 10/group) were gavaged with 100 mg/kg AD and intraperitoneally injected with 300 mg/kg PO daily for 4 weeks.	Quercetin: 200 mg/kg BW/day, i.g.; L. aviarius CML180: 0.2 mL of 1 × 10^8^ CFU/mL bacterial solution, i.g.	↑ *Lactobacillus*; ↓ *Clostridium perfringens*	Increase the hydrophobicity of L. aviarius CML180 and its ability to co-aggregate with *Clostridium perfringens*. Promote the adhesion of L. aviarius CML180 to cells in the intestinal epithelium. Upregulate purine nucleoside-hydrolyzing activity.	[65]
Fisetin	Male C57BL/6J mice (*n* = 6/group) were administrated AD (160 mg/kg) and PO (2400 mg/kg) every other day for 4 weeks.	50 and 100 mg/kg BW/day, p.o.	↑ Epsilonbacteraeota, Bacteroidetes; ↓ Firmicutes	Inhibit gut microbiota-mediated tryptophan metabolism and AHR activation.	[66]
*Camellia japonica* bee pollen polyphenols	Male KM mice (*n* = 10/group) were administered intragastrically with 300 mg/kg PO for 7 days.	2 and 4 g/kg BW/day, i.g.	↑ *Lactobacillus*, *Clostridiaceae*, *Proteobacteria*; ↓ *Firmicutes*, *Bacteroidia.*	Increase SCFAs (acetic acid, butyric acid); provide energy for excretion of uric acid by the cells in the intestinal wall.	[67]
Kidney tea (KT)	Male C57BL/6 mice (*n* = 6/group) were given 300 mg/kg PO (i.p.) for 14 days.	1.6 g/kg BW/day, i.g.	↑ *Roseburia*, *Enterorhabdus*; ↓ *Ileibacterium*	Upregulate the biosynthesis of phenylalanine/tyrosine/tryptophan.	[68]
Astragaloside IV (AST)	Male SD rats (*n* = 6/group) were fed with 10% yeast and 0.15% AD for 4 weeks.	5 and 10 mg/kg BW/day, p.o.; 2 mg/kg BW/day i.p.	↑ *Faecalibacterium*, *Lachnospira*; ↓ *Eubacterium*, *Parabacteroides*, *Clostridium*	Regulate urea metabolism, anti-calcification, and SCFA generation through gut microbiota.	[69]
Astragalus membranaceus ultrafine powder (AMUP)	Pathogen-free male SD rats with intragastric administration of 300 mg/kg PO combined with 10% fructose water feeding for 24 days.	1.5 and 3 g/kg BW/day, i.g.	↑ *Clostridium*, Trichosporonaceae	Decrease the content of bile acid and its downstream metabolites in fecal, and decrease the levels of biomarkers related to immune cells and intestinal inflammatory responses.	[70]
Rare ginsenosides (RGS)	Male KM mice (*n* = 6/group) were injected intraperitoneally with 300 mg/kg/d PO for 35 days.	50, 100 and 200 mg/kg BW/day, p.o.	↑ Firmicutes/Bacteroidetes (F/B) ratio	Upregulate sphingolipid metabolism and pyrimidine metabolism.	[71]
*A. oxyphylla* polysaccharide (AFP)	Male C57 BL/6J mice (*n* = 10/group) were given 50 mg/kg PO (i.g.) and 250 mg/kg AD (i.g.) for 28 days.	100 and 200 mg/kg BW/day, i.g.	↑ *Prevotella*, *Ruminococcus*; ↓ *Parabacteroides*, *Helicobacter*	Regulate the metabolism of pyrimidine, alanine, aspartate, and glutamate.	[72]
Levan	Male SD rats (*n* = 8/group) were given a mixed solution of 1.0 g/kg PO (i.g.) and 0.1 g/kg HX (i.g.) for 3 weeks.	100 and 200 mg/kg BW/day, i.g.	↑ Muribaculaceae, *Faecalibaculum*, *Bifidobacterium*, *Lactobacillus*; *Roseburia* ↓ *Lactobacillus*, *Proteus*	↑ SCFAs, LysoPCs, Pi(38:4), GPlns(18:0/20:4) and Glyceraldehyde; ↓ O-acetylcarnitine, SLPC. Upregulate lipid metabolism, glycerophospholipid metabolism, and immune response.	[73]
Insoluble fiber in Barley Leaf	Male KM mice (*n* = 6/group) received 350 mg/kg/d PO intraperitoneally and 70 mg/kg/d AD by gastric gavage for 14 days.	2.5% (*w*/*w*) in diet, p.o. (≈1.34% insoluble fiber by weight)	↑ *Bacteroides*, *Alloprevotella*, *Eisenbergiella*	Increase fecal SCFAs (acetate, propionate, butyrate).	[74]
Guluronate oligosaccharides (GOS)	Male Balb/c mice (*n* = 11/group) were fed a diet containing 25% yeast and injected intraperitoneally with PO for 4 weeks.	200 mg/kg BW/day, i.g.	↑ Lachnospiraceae_UCG_006, *Ruminococcus*; ↓ *Bilophila*, *Tuzzerella*	Enhance colonic SCFAs; reduce mRNA levels of intestinal GLUT9.	[75,76]
Sporisorium reiliana polysaccharides	SPF C57BL/6 male (*n* = 8/group) mice were given 13% fructose solution for 8 weeks.	50 and 100 mg/kg BW/day, i.g.	↓ Bacteroidetes, Proteobacteria	Decrease the expression of genes involved in glycolysis/gluconeogenesis metabolic pathways and purine metabolism.	[76]
Nuciferine	Male SD adult rats (*n* = 10/group) were orally administered with 250 mg/kg PO.	25 mg/kg BW/day, p.o.	↑ Firmicutes; ↓ Bacteroidetes, Tenericutes	Regulate glycolysis and TCA metabolism; increased glycine, serine, glycerophospholipid in urine and plasma. Reduce intestinal uric acid by downregulating nucleotide metabolism.	[77]
Sulforaphane (SFN)	Male SD rats (*n* = 10/group) were fed with common feedstuffs and 20% yeast with 4% PO for 6 weeks.	10 mg/kg BW/day, i.g.	↑ *Streptomyces*, *Clostridium azureum*, *Clostridium lactis*	Epigenetic modification of Nrf2 and interaction between gut microbiota and epigenetic modification.	[78]
GPAGPR and GPSGRP from *Apostichopus japonicus*	Male C57BL/6 mice (*n* = 5/group) received 200 mg/kg/d of HX (i.g.), 30 mg/kg/d of yeast extract (i.g.) and 250 mg/kg/d of PO (i.g.) for 12 weeks.	10 mg/kg BW/day, i.g.	↑ *Lactobacillus*; ↑ *Escherichia*, *Bilophila*, *Desulfovibrio*	Modulate microbial SCFA, bile acid, and tryptophan metabolism.	[79]
Tuna meat oligopeptides (TMOP)	Male ICR mice (*n* = 8/group) received 200 mg/kg/d HX (i.g.), 30 mg/kg/d yeast extract (i.g.) and 250 mg/kg/d PO (i.g.) for 8 weeks.	50 and 300 mg/kg BW/day, i.g.	↑ *Clostridium*, *Ruminococcus*, *Bifidobacterium*, *Eubacterium*	Increase mRNA and protein level of the occludin and claudin-1.	[80]

i.p.: intraperitoneal injection; i.g.: intragastric gavage; p.o.: oral administration. ↑: increase; ↓: decrease.

**Table 2:** Proposed mechanisms by which natural bioactives regulate intestinal barrier function and inflammation in prevention of hyperuricemia.

Bioactive Compound	Model	Dose	Mode of Mechanism	Reference
Curcumin	Male Wistar rats (*n* = 10/group) were treated with 150 mg/kg AD (i.g.) and 250 mg/kg PO (i.g.) for 4 weeks.	200 mg/kg BW/day, i.g.	Increase protein expression of ZO-1 and occludin in the ileum.	[81]
Ethanol extracts of Torreya grandis seed (EST)	SPF male Kunming mice (*n* = 7/group) received intraperitoneal injections of 300 mg/kg PO for two weeks.	5 mg/kg BW/day, i.g.	Increase protein expression of NPT1 in intestine. Produce SCFAs with immunomodulatory and anti-inflammatory effects on the intestine. Provide colon protection and have a positive association with tight junction proteins.	[82]
Fucoidan	Male C57BL/6J mice (*n* = 10/group) were fed with a high yeast diet (10% *w*/*w* yeast) and given daily intragastric administration of PO (250 mg/kg) and AD (100 mg/kg) for 10 weeks.	150 and 300 mg/kg BW/day, i.g.	Reduce serum lipopolysaccharide and improve the intestinal mucosal barrier function. Increase butyric acid; enhance the expression of ABCG2 via the AMPK/AKT/CREB pathway in ileum.	[83]
Chicory	Male quails (*n* = 8/group) were fed the formulation with added yeast extract powder (15 g/kg) for 60 days.	6.6, 13.3 and 16.7 g/kg BW/day, i.g.	Increase the mRNA and protein expressions of occludin, claudin-1 in small intestine; reduce serum LPS.	[84]
Extracts of *Dendrobium candidum* leaves	Male SD rats (*n* = 8–10/group) were given high-purine diet (0.15% adenine, 10% yeast extract, and 89.85% standard diet) for 5 weeks.	4.375 and 17.5 mg/kg BW/day, i.g.	Increase villus height, reduce crypt depth, enhance barrier function, and suppress the TLR4/NF-κB pathway, attenuating intestinal inflammation and permeability induced by hyperuricemia.	[85]
*Plantaginis Semen polysaccharides* (PSP)	Male SD rats (*n* = 10/group) were gavaged with 100 mg/kg AD and 300 mg/kg PO mixed once daily and the rats were fed food mixed with yeast for 7 weeks.	1.35, 2.7 and 5.4 g/kg BW/day, i.g.	Increase the intestinal tight junction proteins occludin and ZO-1. Inhibit inflammatory cascades and regulate renal uric acid transport proteins.	[86]
Chlorogenic acid (CGA)	Male KM mice (*n* = 10/group) were treated with 300 mg/kg HX (i.g.) and 300 mg/kg PO (i.p.) for 19 days.	30 and 60 mg/kg BW/day, i.g.	Upregulate purine metabolism, glutamate metabolism, increase mRNA expression of ZO-1 and occludin, and reduce mRNA of IL-1β and IL-6 in ileum.	[87]
Leech *Poecilobdella manillensis* total protein extract	KM mice (*n* = 8–10/group) were provided with a high-purine diet and intraperitoneal injection 200 μL PO for 7 consecutive days.	49 mg/kg BW/day, i.g.	Increase ZO-1 protein expression in jejunal tissue, regulated sphingolipid metabolism, and galactose metabolism pathways.	[88]
Allicin	Male SD rats (*n* = 10/group) with PO (750 mg/kg, i.g.) and yeast (10 g/kg, i.g.) induced HUA and intra-articular MSU (0.15 mL, 25 mg/mL) induced gouty arthritis; treated for 7 days.	15 and 30 mg/kg BW/day, i.g.	Decrease serum uric acid and XOD activity; suppress synovial oxidative stress (↓ ROS, ↓ MDA; ↑ SOD, ↑ GSH); inhibit NLRP3 inflammasome activation (↓ NLRP3, ASC, caspase-1, IL-1β) and pro-inflammatory cytokines (↓ TNF-α, IL-8, IL-18) in synovial tissue; restore gut microbiota diversity and increase *Lactobacillus* abundance; and elevate butyric acid levels.	[89]

i.p.: intraperitoneal injection; i.g.: intragastric gavage; p.o.: oral administration. ↑: increase; ↓: decrease.

**Table 3:** Proposed mechanisms by which natural bioactives regulate intestinal uric acid transporters in prevention of hyperuricemia.

Bioactive Compound	Model	Dose	Mode of Mechanism	Reference
Eupatilin	Male SD rats (*n* = 8/group) were given PO and HX (both 300 mg/kg, p.o.) for 14 days.	20, 40 and 80 mg/kg BW/day, p.o.	Inhibit activities of the ADA and XOD enzyme, decrease expression of GLUT9 and URAT1, and increase expression ABCG2 in ileum.	[31]
Mangiferin	The KM mice (*n* = 8–10/group) with intraperitoneal injection of 300 mg/kg PO for 7 days.	3, 6 and 12 mg/kg BW/day, i.g.	Increase protein expression of ABCG2 and inhibit the protein expression of GLUT9 markedly in a dose-dependent manner in the intestine.	[90]
Berberine	Male SD male rats (*n* = 10–12/group) were given PO (1500 mg/kg/day with food) for 7 days.	100 mg/kg BW/day, p.o.	Upregulate N-glycan biosynthesis, starch/sucrose metabolism, sphingolipid metabolism, increase the level of ABCG2, and decrease the level of urate transport Galectin-9 in the colon.	[91]
Dioscin (Tiogenin and Diosgenin)	Male SD rats (*n* = 8/group) and KM mice (*n* = 8–10/group) were intragastrically administrated with 300 mg/kg PO.	25 and 50 mg/kg BW/day, p.o.	Upregulate ABCG2-mediated UA efflux.	[92]
Ferulic acid (FA)	Male SD rats (*n* = 10/group) induced by a high-fructose/fat diet (18.9 kJ g−1, 18% fructose and 20% lard) for 20 weeks.	0.05% and 0.1% (50 mg and 100 mg per 100 g diet, p.o.	Reduce intestinal ABCG2 mRNA levels, and increase *Slc2a9* and *Slc22a13* in duodenum, jejunum, and ileum.	[93]

i.p.: intraperitoneal injection; i.g.: intragastric gavage; p.o.: oral administration.

**Table 4:** Proposed mechanisms by which natural bioactives treat hyperuricemia through the synergistic action of multiple targets.

Bioactive Compound	Model	Dose	Microbiota Findings	Intestinal Barrier	Intestinal Inflammation	Uric Acid Transporters	Reference
Inulin-type fructans	*Uox*-knockout mouse model (C57BL/6J genetic background, *n* = 8/group).	9.5 g/kg BW/day (i.g.) last 7 weeks	↑ *Akkermansia Bifidobacterium*, *Parasutterella* and *Ruminococcus*; ↓ *Bacteroides*	Increase intestinal TJ proteins (ZO-1 and occludin); reduce serum DAO & d-LAC	Lower serum and ileum IL-1β, IL-6, TNF-α; reduce serum LPS	Upregulate the mRNA level of ABCG2 in jejunum and ileum.	[94]
Oleanolic acid (OA)	Male C57BL/6J mice (*n* = 8/group) were given 250 mg/kg/d PO (i.g.) and 250 mg/kg/d HX (i.g.) for 12 weeks.	25, 50 and 100 mg/kg BW/day, i.g.	↑ *Enterorhabdus*, *norank_f__norank_o__* *Clostridia_*UCG-014, Lachnospiraceae_ NK4A136_group; ↓ *Bacteroides*, *Staphylococcus*	Increase the villus height-to-crypt depth (V/C) ratio, and upregulate the protein expressions of occludin, claudin-1 and ZO-1	Reduce renal IL-6, TNF-α, TLR4; FMT reproduced anti-inflammatory phenotype	Increase intestinal ABCG2 protein expression, and decrease intestinal GLUT9 protein expression.	[95]
Pro-Glu-Trp (PEW) from Whey Protein	Male SD rats (*n* = 6/group) were given gavage of 500 mg/kg PO and 500 mg/kg HX for 28 days.	30 and 60 mg/kg BW/day, i.g.	↑ Muribaculaceae, *Lactobacillus*, *Ruminococcus;* ↓ *Bacteroides*, *Alloprevotella*, *Desulfovibrio*	Upregulate the expression of tight junction protein occludin and ZO-1; restore villus height/crypt depth	Lower serum IL-1β, IL-6, TNF-α and LPS	Increase ABCG2 and GLUT9 protein expressions in jejunum and ileum.	[96]

i.p.: intraperitoneal injection; i.g.: intragastric gavage; p.o.: oral administration; ↑: increase; ↓: decrease.

## Data Availability

Data sharing is not applicable to this article as no new data were created or analyzed in this study.

## Abbreviations

The following abbreviations are used in this manuscript:

UA	Uric Acid
HUA	Hyperuricemia
XOD	Xanthine Oxidase
ADA	Adenosine Deaminase
AMP	Adenosine Monophosphate
ABCG2	ATP-binding cassette subfamily G2
GLUT9	Glucose Transporter 9
SLC2A9	Solute Carrier Family 2 Member 9
URAT1	Urate Anion Transporter 1
OAT	Organic Anion Transporter
SCFAs	Short-Chain Fatty Acids
TJ	Tight Junction
ZO-1	Zonula Occludens-1
DAO	Diamine Oxidase
D-LAC	D-Lactic Acid
LPS	Lipopolysaccharide
TLR4	Toll-like receptor 4
NF-κB	Nuclear factor kappa-light-chain-enhancer of activated B cells
IL-1β	Interleukin-1 Beta
IL-6	Interleukin-6
TNF-α	Tumor Necrosis Factor-Alpha
GALT	Gut-Associated Lymphoid Tissue
NLRP3	NOD-like Receptor Family Pyrin Domain Containing 3
MSU	Monosodium Urate
SNP	Single nucleotide polymorphism
FMT	Fecal microbiota transplantation
Nrf2	Nuclear Factor Erythroid 2-Related Factor 2
CKD	Chronic kidney disease
AHR	Aryl Hydrocarbon Receptor
PPARγ	Peroxisome Proliferator-Activated Receptor Gamma
TMAO	Trimethylamine N-Oxide
OAT10	Organic Anion Transporter 10
KO	Knockout
WT	Wild Type
V/C	Villus Height-to-Crypt Depth Ratio
AMPK	AMP-activated Protein Kinase
AKT	Protein Kinase B
CREB	cAMP Response Element-Binding Protein
SPF	Specific Pathogen-Free
SD	Sprague–Dawley
KM	Kunming
C57BL/6J	C57 Black 6 Mouse Strain
ICR	Institute of Cancer Research
HFD	High-Fat Diet
PO	Potassium Oxonate
HX	Hypoxanthine
AD	Adenine
BW	Body Weight
CFU	Colony Forming Units
i.p.	Intraperitoneal injection
i.g.	Intragastric gavage
p.o.	Per os (oral administration)
mRNA	Messenger RNA
NMR	Nuclear Magnetic Resonance
LC-MS	Liquid Chromatography–Mass Spectrometry
